# Diagnosis-dependent metabolic reprogramming of follicular fluid

**DOI:** 10.1186/s12958-026-01579-0

**Published:** 2026-06-19

**Authors:** Csilla Kurdi, Dávid Hesszenberger, Dávid Csabai, Anikó Lajtai, Ágnes Lakatos, Krisztina Gödöny, Péter Mauchart, Ákos Várnagy, Gábor L. Kovács, Tamás Kőszegi

**Affiliations:** 1 Molecular Medicine Research Group, Szentágothai Research Center, University of Pécs, Ifjúság u. 20, Pécs, H-7624 Hungary; 2 National Laboratory on Human Reproduction, University of Pécs, Pécs, H-7624 Hungary; 3 Department of Laboratory Medicine, Medical School, University of Pécs, Pécs, H-7624 Hungary; 4 Department of Obstetrics and Gynecology, Medical School, University of Pécs, Pécs, H-7624 Hungary; 5 MTA-PTE Human Reproduction Scientific Research Group, University of Pécs, Pécs, H-7624, Hungary

**Keywords:** Follicular fluid, Amino acids, Insulin resistance, Endometriosis, Thyroid dysfunction, Metabolomics, In vitro fertilization

## Abstract

**Background:**

The follicular fluid (FF) microenvironment plays a critical role in oocyte maturation and embryo development, reflecting local ovarian activity and systemic metabolic status. While metabolic alterations in FF have been described in different diseases, comparative analyses across different infertility-related disorders remain limited.

**Objective:**

This study aimed to characterize and compare amino acid profiles in FF from patients undergoing in vitro fertilization (IVF) with insulin resistance (IR), endometriosis (EM), thyroid dysfunction (TD) and to identify disease-specific metabolic signatures.

**Methods:**

We analyzed 171 FF samples using targeted ultra-high-performance liquid chromatography. The twenty proteogenic amino acids were quantified and analyzed using univariate and multivariate statistical analyses, including Kruskal-Wallis testing with post-hoc correction, generalized linear modeling adjusted for BMI, principal component analysis (PCA) and pathway enrichment analysis.

**Results:**

PCA revealed that the global amino acid composition of FF was largely conserved across all groups. However, disease status had a statistically significant but moderate effect on the overall amino acid profile (PERMANOVA, R²=0.081, *p* = 0.001). After adjusting for BMI, IR was associated with decreased glycine and arginine levels and TD was associated with lower histidine, tryptophan and lysine concentrations. In contrast, EM was characterized by a selective increase in the histidine content. Pathway analysis revealed alterations in branched-chain amino acid (BCAA) metabolism in IR group and broader disruptions in central amino acid metabolism in TD group. Multivariate and ROC analyses indicated limited discriminative performance of individual amino acids (AUC ≤ 0.71), suggesting that metabolic alterations are subtle and distributed across multiple pathways.

**Conclusions:**

FF amino acid composition is tightly regulated but distinct disease-specific metabolic alterations can be detected in IR, EM and TD. These changes are independent of BMI and reflect coordinated alterations in the metabolism of different amino acids rather than strong individual biomarkers. These results show the sensitivity of the follicular environment to overall metabolic health and support the idea of using multivariable metabolic patterns to better understand reproductive dysfunction.

**Supplementary Information:**

The online version contains supplementary material available at https://doi.org/10.1186/s12958-026-01579-0.

## Introduction

Successful oocyte maturation and embryo development depend on a finely tuned microenvironment within the ovarian follicle [1]. The follicular fluid (FF) surrounding the oocyte not only nourishes the gamete but also reflects the dynamic interplay between local ovarian activity and systemic physiological states [2, 3]. Composed of metabolites secreted by granulosa and theca cells, as well as circulating components crossing the blood-follicle barrier, FF represents a picture of both the follicular and whole-body physiology at the time of oocyte retrieval [3, 4]. 

Among its constituents, amino acids play multifaceted roles that extend far beyond serving as building blocks for protein synthesis. They provide energy, regulate osmotic balance and act as precursors for signaling molecules and antioxidants such as glutathione [5]. Emerging evidence indicates that alterations in FF amino acid composition can influence oocyte competence, fertilization rates and early embryo development, suggesting that the follicular amino acid milieu serves as a sensitive indicator of reproductive health [6]. 

The three conditions investigated in this study (IR, EM and TD) were selected to represent mechanistically distinct pathophysiological categories that are frequently encountered in the IVF population. Endocrine and metabolic disorders, such as IR and TD, and inflammatory conditions such as EM, are frequently associated with infertility and suboptimal assisted reproductive technology (ART) outcomes [7–9]. IR represents a systemic metabolic disorder characterized by glucose and amino acid utilization within the follicle, potentially limiting nutrient availability for developing oocytes; [10] EM is a chronic inflammatory condition associated with oxidative stress and local immune dysregulation that may alter metabolic signaling in FF; [11] and TD represents an endocrine disorder whose hormonal imbalance can profoundly affect ovarian function and the local follicular environment through board systemic metabolic consequences [12]. This selection was deliberate: by examining conditions that differ fundamentally in their etiology and primary site of action, we wanted to determine whether the follicular fluid amino acid microenvironment carries disease-specific metabolic signatures or whether infertility-associated conditions converge on shared biochemical alterations regardless of their underlying pathophysiology. Despite the clinical significance of these conditions, the mechanism by which they uniquely reprogram the FF amino acid landscape remain unclear. Previous studies have often examined single disorders [13–15] or global metabolic trends, leaving a gap in our understanding of diagnosis-specific alterations. Recent advances in ultra-high-performance chromatography (UHPLC) have enabled the precise and comprehensive quantification of all proteogenic amino acids, offering an opportunity to map disease-associated metabolic shifts with high resolution.

In this study, we aimed to characterize FF amino acid profiles in patients with IR, EM and TD compared with healthy controls. While amino acid alterations in EM have been partially addressed in our previous work [14] the present study extends this analysis by simultaneously comparing three mechanistically distinct pathophysiological conditions within a unified FF amino acid profiling framework, including substantially larger study groups. To the best of our knowledge, this is the first study to enable direct cross-disease comparison of diagnosis-specific metabolic signatures across IR, EM and TD. By integrating multivariate statistical modeling, pathway and biomarker analysis and correlation with clinical parameters, we aimed to uncover diagnosis-dependent metabolic signatures. Understanding these patterns may provide mechanistic information on ovarian dysfunction, highlight potential biomarkers for oocyte competence and ultimately inform personalized strategies to optimize ART outcomes.

## Materials and methods

### Study population

This study was performed between July 2025 and February 2026 at the Department of Obstetrics and Gynecology and at the Szentágothai Research Center, both at the University of Pécs, Hungary. All patients received detailed information of the study protocol, and a written informed consent was obtained prior to participation. Patients under 18 years and those who were unable to provide or withdrew the consent were excluded from the study. The study protocol was approved by the Regional Research Ethics Committee of the University of Pécs (No. 4327.316–2900/KK15/2011) in accordance with the 7th revision of the Declaration of Helsinki (2013). Follicular fluid samples were obtained from women undergoing oocyte retrieval as part of their routine assisted reproduction procedure. Based on clinical diagnosis and medical history, the patients were categorized into four groups: control, IR, EM and TD.

The control group consisted of patients without known metabolic or endocrine disorders affecting reproductive function. The participants of the IR group were diagnosed by endocrinologists based on the homeostatic model assessment of insulin resistance (HOMA-IR) formula; patients with diabetes mellitus or polycystic ovary syndrome were excluded from this group. The EM group comprised patients with stage III EM confirmed by ultrasound who had undergone surgical treatment prior IVF. The TD group included patients with a documented history of hypothyroidism with elevated TSH levels who were receiving thyroid hormone replacement therapy. Although all TD patients were biochemically treated at the time of the collection, further subclassification by etiology was not performed.

Relevant clinical parameters, including age and body mass index (BMI) were recorded for all participants. These variables were considered in a subsequent statistical analysis to account for potential confounding effects. Fertilization rate was calculated as the number of fertilized oocytes divided by the total number of inseminated oocytes.

### Follicular fluid collection

Follicular fluid samples were collected during transvaginal ultrasound-guided oocyte retrieval following controlled ovarian hyperstimulation as a part of the IVF procedure. For each patient, FF obtained from all aspirated follicles during the retrieval procedure was pooled to obtain a representative sample of the overall follicular environment, regardless of individual follicle maturity status. Aspirated FF was carefully inspected to avoid visible blood contamination. Samples were centrifuged (6700× g for 10 min at room temperature) to remove cellular debris and the supernatant was aliquoted and stored at -80 °C until further analysis.

For each patient, FF obtained from all aspirated follicles during the retrieval procedure was pooled to obtain a representative sample of the overall follicular microenvironment, regardless of individual follicle maturity status.

### Amino acid quantification

Targeted quantification of twenty proteinogenic amino acids in FF samples were performed using ultra-high-performance liquid chromatography (Shimadzu Nexera X2 UHPLC System) with fluorescence detection (RF-20 A XS, both from Shimadzu Europa GmbH Duisburg, Germany), after protein precipitation and amino acid derivatization.

Proteins were precipitated by adding 300 µL ice-cold acetonitrile to the samples, followed by centrifugation for 4 min at 6100 g (ScanSpeed Mini, Labogene, Allerod, Denmark). The supernatant was diluted with 600 µL phosphate buffer and filtered (Millex^®^ GV 4 mm Durapore PVDF 0.22 μm, Merck KGaA, Darmstadt, Germany) prior to analysis. The amino acids were then derivatized using ortho-phtalaldehyde (OPA, ≥ 99% HPLC grade, Merck KGaA, Darmstadt, Germany) in the presence of 3-mercaptopropionic acid (MPA, ≥ 99.0%, HPLC grade, Merck KGaA, Darmstadt, Germany), while proline was derivatized using 9-fluorenylmethylcarbonyl chloride (FMOC, ≥ 99.0% (HPLC grade, Merck KGaA, Darmstadt, Germany). L-norvaline (250 µmol/L L-Norvaline, Merck, KgaA, Darmstadt, Germany) was used as an internal standard for quantitative analysis.

Chromatographic separation was performed on a reverse-phase C18 column (100 × 3.0 mm Kinetex 2.6 μm EVO C18 100Å (Phenomenex, Torrance, CA, USA) using gradient mobile phase consisting of 20 mmol/L phosphate buffer (A) and a 40:45:15 acetonitrile: methanol: water solution (B). The flow rate was 1.3 mL/min and the column temperature was maintained at 27 °C. The total running time was 15.1 min. All amino acids except proline were detected using a fluorescence detector (RF-20 A XS, Shimadzu) at 350 nm excitation and 450 nm emission. Proline was measured separately at 266 nm excitation and 305 nm emission. The analysis was performed by Shimadzu LabSolutions 5.97 SP1 software. The amino acids were identified based on retention times and quantified using the peak area relative to the internal standard. All samples were analyzed in duplicate and final concentrations were calculated as the mean of the two parallel measurements.

### Data preprocessing and statistical analysis

Before the statistical analysis, amino acid concentration data were subjected to median normalization, followed by log10 transformation and auto-scaling to reduce systematic variation and enhance comparability across samples. Data distribution and normality were evaluated using the Shapiro-Wilk test. Most variables did not follow normal distribution so non-parametric statistical methods were applied.

#### Univariate and post-hoc analysis

Kruskal-Wallis test was used to initially evaluate the differences in amino acid concentration in the four study groups. For those amino acids that showed significant global differences, post-hoc pairwise comparisons were performed using Dunn’s test. In order to control the family-wise error rate, p-values were adjusted using Bonferroni corrections. The magnitude and direction of metabolic differences between disease groups and the control group were expressed as log 2-fold change (log2FC).

#### Adjustment for potential confounding factors

Given the observed differences in clinical characteristics among the study groups, particularly body mass index (BMI), generalized linear models (GZLM) were applied to evaluate whether the identified metabolic alterations remained significant after adjusting for BMI as a potential confounding factor.

#### Multivariate analysis

Multivariate analyses were performed to explore global metabolic patterns and identify metabolites contributing to group separation. Unsupervised principal component analysis (PCA) was first applied to visualize overall variation in amino acid profiles across samples. Partial least squares-discriminant analysis (PLS-DA) was performed to identify amino acids contributing to group separation. Models were constructed using autoscaled data and the optimal number of components were determined by cross-validation. Model performance was evaluated using R^2^ (explained variance) and Q^2^ (predictive ability) metrics. To assess model robustness and avoid overfitting, permutation testing was conducted with 1000 iterations.

Variable importance in projection (VIP) scores were calculated to estimate the contribution of each amino acid to the model. Metabolites with VIP > 1 were considered relevant contributors to group discrimination.

#### Random forest analysis

Random Forest (RF) classification was applied using the MetaboAnalyst platform to rank amino acids according to their importance in distinguishing between disease groups and controls. The RF models were constructed using normalized and scaled dataset with disease group as the response variable and amino acid concentration as predictors.

Model training was performed using an ensemble of decision trees (*n* = 500) and feature importance was assessed based on the mean decrease in accuracy. Model performance was internally evaluated using out-of-bag error estimation. The resulting importance score were used to identify key metabolites contributing to group classification.

#### Pathway enrichment analysis

To investigate metabolic pathways potentially associated with the observed amino acid alterations, pathway enrichment analysis was performed using Metaboanalyst platform. Identified metabolites were mapped to metabolic pathways based on the Kyoto Encyclopedia of Genes and Genomes (KEGG) database. Enrichment significance was evaluated using over-representation analysis combined with pathway topology analysis and the results were reported as raw p-values, false discovery rate (FDR) and pathway impact scores.

#### Biomarker analysis

Receiver operating characteristics (ROC) curve analysis was performed to evaluate the discriminatory ability of each amino acid. As a measure of diagnostic performance, the area under curve (AUC) was calculated. For multivariate ROC analysis, classification models were constructed using combinations of amino acids and model performance was assessed based on AUC values. Due to the relatively small metabolic differences between groups, ROC analyses were interpreted with caution, focusing on overall trends than single, high-performing biomarkers.

## Results

### Patient characteristics

In this study 171 FF samples were measured and the 20 main proteogenic amino acids were determined. The samples were classified based on the existing diseases and four groups were identified namely control group (*n* = 98), EM group (*n* = 19), IR group (*n* = 21) and TD group (*n* = 33).

The clinical characteristics of the study groups are summarized in Table 1. The mean age of patients was comparable across all groups, ranging from 33.3 years in the IR group to 34.97 years in the TD group. Body mass index differed between groups, with the highest values observed in the IR group, while patients with EM showed the lowest BMI. These findings are consistent with current literature, which frequently reports a strong correlation between insulin resistance and elevated BMI [16] and a strong correlation between lower BMI and EM [17].

**Table 1:** General statistics of patients involved in the study

	Control group	EM group	IR group	TD group
Age	34.20 ± 5.26	34.28 ± 4.78	33.33 ± 5.78	34.97 ± 7.48
BMI	25.06 ± 5.22	22.09 ± 5.17	30.33 ± 5.08	24.47 ± 4.04
Number of oocytes retrieved	10.90 ± 7.12	11.39 ± 9.18	15.57 ± 6.80	11.21 ± 5.18
Number of mature oocytes (MII)	6.50 ± 5.33	6.00 ± 4.69	9.04 ± 5.56	7.30 ± 6.55
Number of inseminated oocytes	9.92 ± 6.80	10.11 ± 9.02	14.52 ± 6.82	10.49 ± 5.69
Number of fertilized mature oocytes	3.93 ± 3.53	4.17 ± 2.87	6.00 ± 4.49	6.45 ± 4.44
Fertility rate (%)	38.92 ± 27.55	48.03 ± 25.88	30.40 ± 28.01	57.54 ± 27.74
Number of blastocysts	1.75 ± 2.58	1.67 ± 2.81	2.90 ± 3.71	2.82 ± 3.07

The mean number of retrieved oocytes was similar in the control, EM, and TD groups, whereas a higher number was observed in the IR group. A similar trend was observed for the number of mature (MII) oocytes and inseminated oocytes, which were also highest in the IR group. The number of fertilized mature oocytes ranged from 3.93 in the control group to 6.45 in the TD group. Fertilization rates showed variability among the groups, with the lowest mean value in the IR group and the highest in the TD group. The mean number of blastocysts formed per patient ranged from 1.67 in the EM group to 2.9 in the IR group.

### Global amino acid profile of FF

The overall amino acid composition of FF was evaluated across all samples and summarized in Table 2. Among the 20 amino acids measured, glutamine showed the highest concentration, followed by alanine, glycine, proline and valine. Lower concentrations were detected for several amino acids including aspartate, methionine, cysteine and isoleucine. The relatively wide range of concentrations reflects the diverse metabolic roles of amino acids within the follicular microenvironment.

**Table 2:** Mean concentrations of amino acids in FF samples

	Mean ± SD
Aspartate	6.67 ± 7.78
Glutamate	63.72 ± 27.12
Asparagine	30.28 ± 7.62
Serine	50.01 ± 15.73
Glutamine	347.70 ± 78.51
Histidine	48.93 ± 15.65
Glycine	151.61 ± 46.20
Threonine	103.04 ± 32.29
Arginine	31.37 ± 11.12
Alanine	230.81 ± 63.09
Tyrosine	28.80 ± 9.54
Cysteine	21.26 ± 8.22
Valine	127.61 ± 40.71
Methionine	16.34 ± 6.67
Tryptophan	41.56 ± 14.68
Phenylalanine	37.00 ± 8.66
Isoleucine	28.05 ± 10.10
Leucine	51.23 ± 18.48
Lysine	75.09 ± 31.67
Proline	143.37 ± 58.67

Values are presented as mean ± standard deviation (SD) and expressed in µmol/L

### Global metabolic differences between groups

#### Principal component analysis

**Fig. 1:**
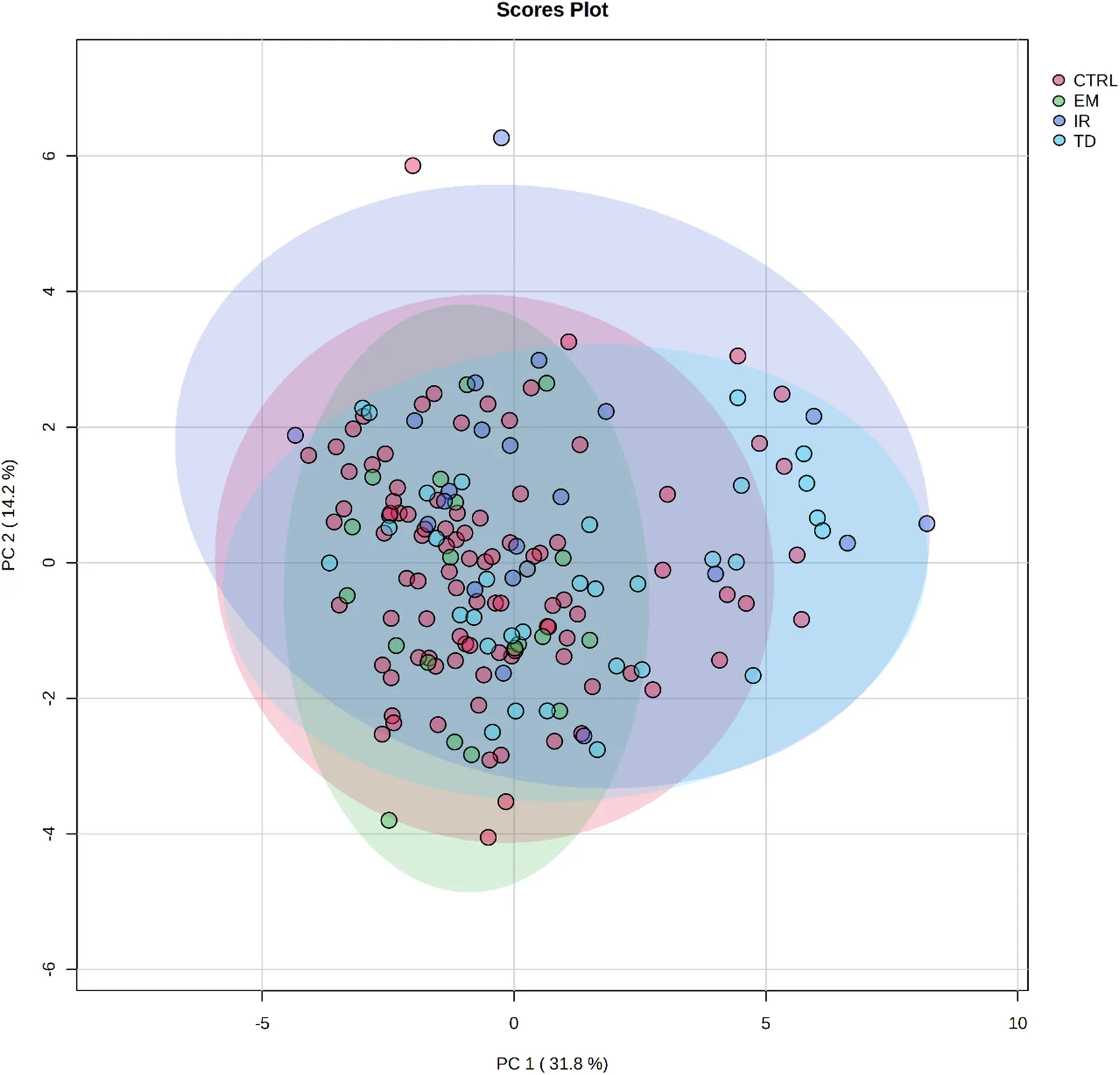
PCA score plot based on FF amino acid concentrations. Samples are grouped according to clinical diagnosis: control, IR, EM, and TD

To explore global differences in FF amino acid composition among the study groups, PCA was performed using the concentrations of the 20 analyzed amino acids. The first two principal components explained 31.8% and 14.2% of the total variance. Together, the first two components explained 46% of the total variance. The PCA score plot (Fig. 1) revealed a substantial overlap between the four groups, suggesting that the overall amino acid composition of the FF is largely conserved across patients.

Despite this overlap, subtle trends in group distribution were observed. Samples from the IR and TD groups showed a tendency to shift along the PC1 relative to the control group, suggesting coordinated alterations in amino acid metabolism associated with these conditions. In contrast, samples from the EM group largely overlapped with the control group, indicating the absence of a pronounced global metabolic shift in this dataset. 

To further interpret the PCA results, the contribution of individual amino acids to the first two principal components was examined (Supplementary Table 1). PC1 was driven by a broad range of amino acids, with the highest contributions from alanine (10.4%), glutamine (8.7%), phenylalanine (7.5%) and threonine (7.3%), indicating that this component reflects overall variation in amino acid abundance. In contrast, PC2 was strongly dominated by BCAAs, including leucine (23.5%), isoleucine (20.2%) and valine (17.1%) which together accounted for approximately 60% of the total contribution. This pattern suggests that coordinated variation in BCAA metabolism represents a major source of metabolic variability in the dataset.

#### Permutational multivariate analysis of variance

To statistically evaluate whether disease status has an influence on the overall amino acid profile of FF, a permutational multivariate analysis of variance (PERMANOVA) was performed. The analysis revealed that the disease status has a significant effect on the overall amino acid profile (F = 4.906, R^2^ =0.081, *p* = 0.001), although it explains only 8.1% of the total variability, indicates a relatively small effect size. Since PERMANOVA test may be sensitive to differences in group dispersion this result should be interpreted in the context of the relatively small effect size and the substantial overlap observed in the PCA.

Together with the PCA results, these findings indicate that disease-associated metabolic alterations in FF are relatively small and arise from coordinated changes across multiple amino acids rather than large shifts in individual metabolite concentrations.

### Disease-associated changes in individual amino acids

To identify individual amino acids that may contribute to the disease-associated metabolic differences, a Kruskal-Wallis test was performed to compare the concentrations of the 20 amino acids measured across the four study groups. Initially, several amino acids, including histidine, glycine, tryptophan and lysine showed significant differences (*p* < 0.05). To account for multiple comparisons and reduce the risk of false-positive findings, we applied a Bonferroni correction. Post-hoc pairwise comparisons using Dunn’s test revealed that these global differences were driven by specific metabolic shifts between patient groups. Notably, histidine (adjusted *p* = 0.008) and lysine (adjusted *p* = 0.027) levels remained significantly different between the TD and EM groups. Furthermore, glycine demonstrated a significant metabolic shift in the IR group compared with controls (adjusted *p* = 0.023).

Several additional amino acids exhibited trends that approached statistical significance, such as arginine (adjusted *p* = 0.086) and tryptophan (adjusted *p* = 0.07), both observed in the comparison between TD and EM patients. These findings indicate that thyroid disorders and insulin resistance are associated with selected differences in FF amino acid composition. (Table 3).

**Table 3:** Amino acids showing significant differences among the study groups

Amino acid	Krustal-Wallis	Dunn’s test	Adj.sign.
Arg	0.107	TD-EM	0.086
Hist	0.012	TD-EM	0.008
Gly	0.022	IR-CTRL	0.023
Try	0.044	TD-EM	0.070
Lys	0.019	TD-EM	0.027

Global differences were evaluated using the Kruskal–Wallis test, followed by Dunn’s post-hoc pairwise comparisons with Bonferroni correction

Adjusted *p*-values indicate statistically significant differences between the specified groups

### Multivariate adjustment for clinical confounders

To evaluate the independent effect of each pathological condition on the FF amino acid profile, a Generalized Linear Model (GZLM) was used. This analysis was specifically designed to separate the metabolic influence of the diseases from the potential confounding effect of BMI with the healthy control group, serving as a reference category. The results are shown in Table 4. Despite the statistical significance of this adjusted model, the overall magnitude of these effects remains modest. This suggests that the observed associations represent relatively small alterations in the amino acid profile.

**Table 4:** GZLM results for amino acids showing significant associations with disease groups after adjustment for BMI

Amino Acid	Disease group effect (*p*)	BMI effect	Dominant group (vs. control)	Effect (B)
Arginine	0.018	0.737	IR	-6.725
Histidine	0.030	0.587	TD	-6.845
Histidine	0.039	0.587	EM	8.228
Glycine	0.032	0.555	IR	-25.77
Tryptophan	0.019	0.994	TD	-7.063
Lysine	0.030	0.342	TD	-13.935

The model evaluated the effect of disease group and BMI on amino acid concentrations. The dominant group indicates the patient group showing the strongest deviation from the control group. Effect size is represented by the regression coefficient (B).

The adjusted models confirmed that the most prominent metabolic shifts identified in the study were robust and independent of the patients’ body mass. Regarding the IR group, the analysis revealed a significant disease- specific depletion of key amino acids, specifically, glycine levels were markedly lower compared to controls (*p* = 0.032, B=-25.77) and arginine concentrations also showed a significant independent decrease (*p* = 0.018, B=-6.725). In both cases, BMI did not act as a significant predictor (*p* = 0.555 and *p* = 0.737), suggesting that these alterations are intrinsic to the pathophysiology of IR.

Patients in the TD group exhibited the most extensive metabolic reprogramming within the follicular environment. This group was characterized by a significant reduction in the concentrations of histidine (*p* = 0.030, B=-6.845), tryptophan (*p* = 0.019, B=-7.06) and lysine (*p* = 0.030, B=-13.935). The lack of association between these amino acids and BMI (all *p* > 0.30) suggests that the observed alterations are independent of BMI and may reflect TD-related changes in the follicular amino acid microenvironment.

In contrast, the metabolic signature of the EM group was more specific, showing significant elevation in histidine levels compared to the controls (*p* = 0.039, B = 8.228). While other amino acids showed nominal variations in this group, only the shift in histidine remained statistically significant after adjusting for clinical confounders. 

### Multivariate discrimination analysis

#### Partial least squares-discriminant analysis

**Fig. 2:**
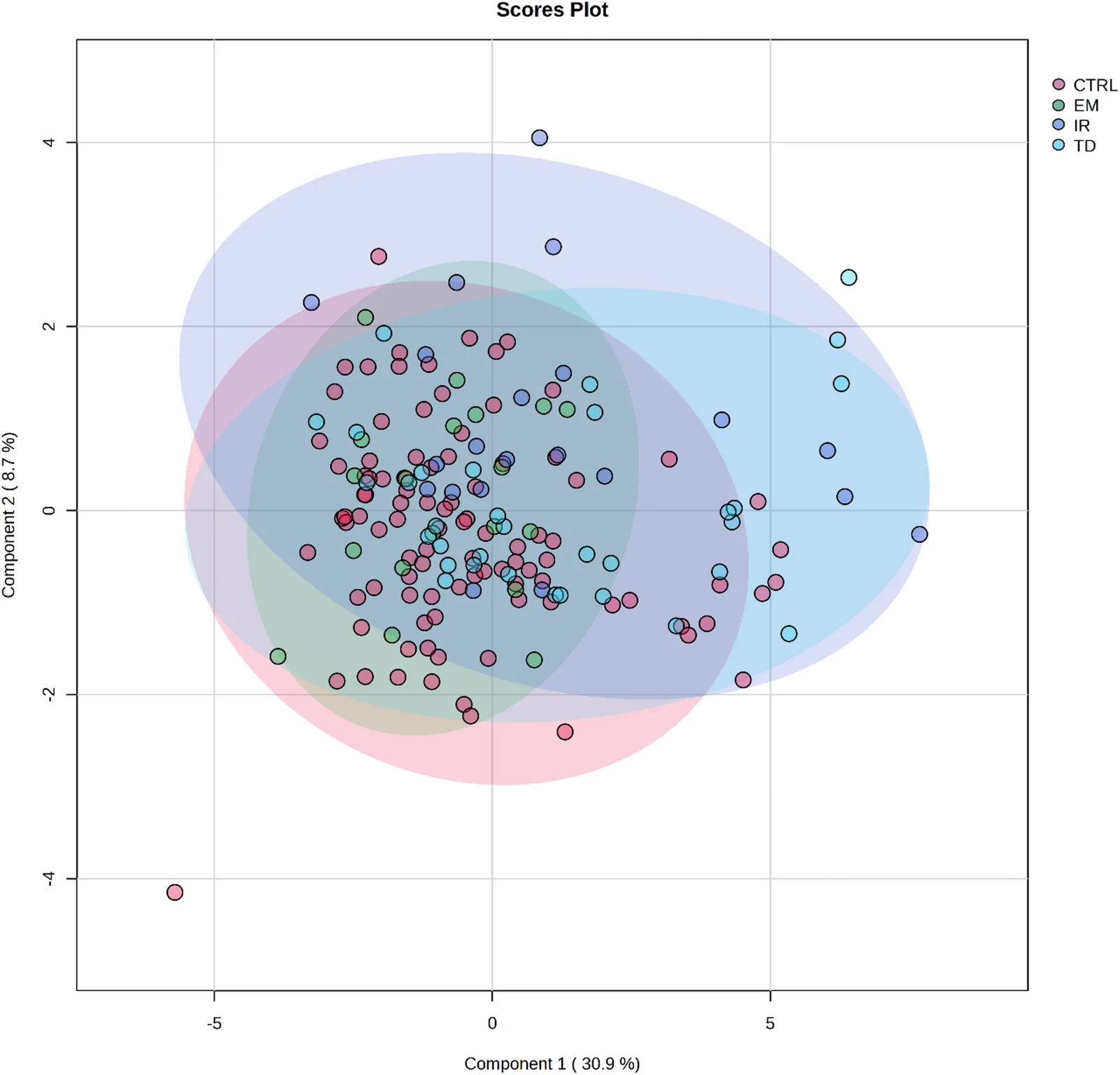
PLS-DA score plot based on FF amino acid concentrations in the four study groups. Samples are colored according to diagnosis: CTRL, IR, EM, and TD. Ellipses indicate group clustering patterns

To further explore the multivariate structure of the amino acid dataset and identify metabolites that contribute to the group separation, partial least squares-discriminant analysis (PLS-DA) was performed. As a supervised method, PLS-DA maximizes class separation and may find group structures that PCA did not captured, however, the results of this analysis require cautious interpretation. The PLS-DA score plot (presented on Fig. 2) showed a partial clustering of samples according to disease status, with the IR and TD groups displaying the most distinct tendencies, while the EM samples largely overlapped with the control group. These observations were consistent with the PCA results, suggesting that the metabolic alterations associated with IR and TD may contribute more strongly to the overall variability of the FF amino acid profile than those observed in EM.

Model quality was assessed using R2 and Q2 statistics derived from cross-validation. Q2 values represent the predictive ability of the model and were used as the primary indicator of the robustness of the model. The first component showed a slightly positive Q^2^ value, indicating limited predictive capacity for the model. To further assess model robustness, a permutation test with 1000 permutations was performed. The empirical p-value was not significant (*p* = 0.677), indicating that the observed group separation was not stronger than expected by chance. These results suggest that although the PLS-DA visualization showed mild clustering patterns, the overall discriminative power of the supervised model remains limited. 

#### Variable importance in projection scores

The VIP scores were used to identify the metabolites contributing most strongly to group separation. The highest VIP scores were observed for threonine (VIP > 1.72), lysine (VIP > 1.47) and alanine (VIP > 1.4), suggesting that these amino acids contribute most strongly to the observed multivariate patterns.

**Fig. 3:**
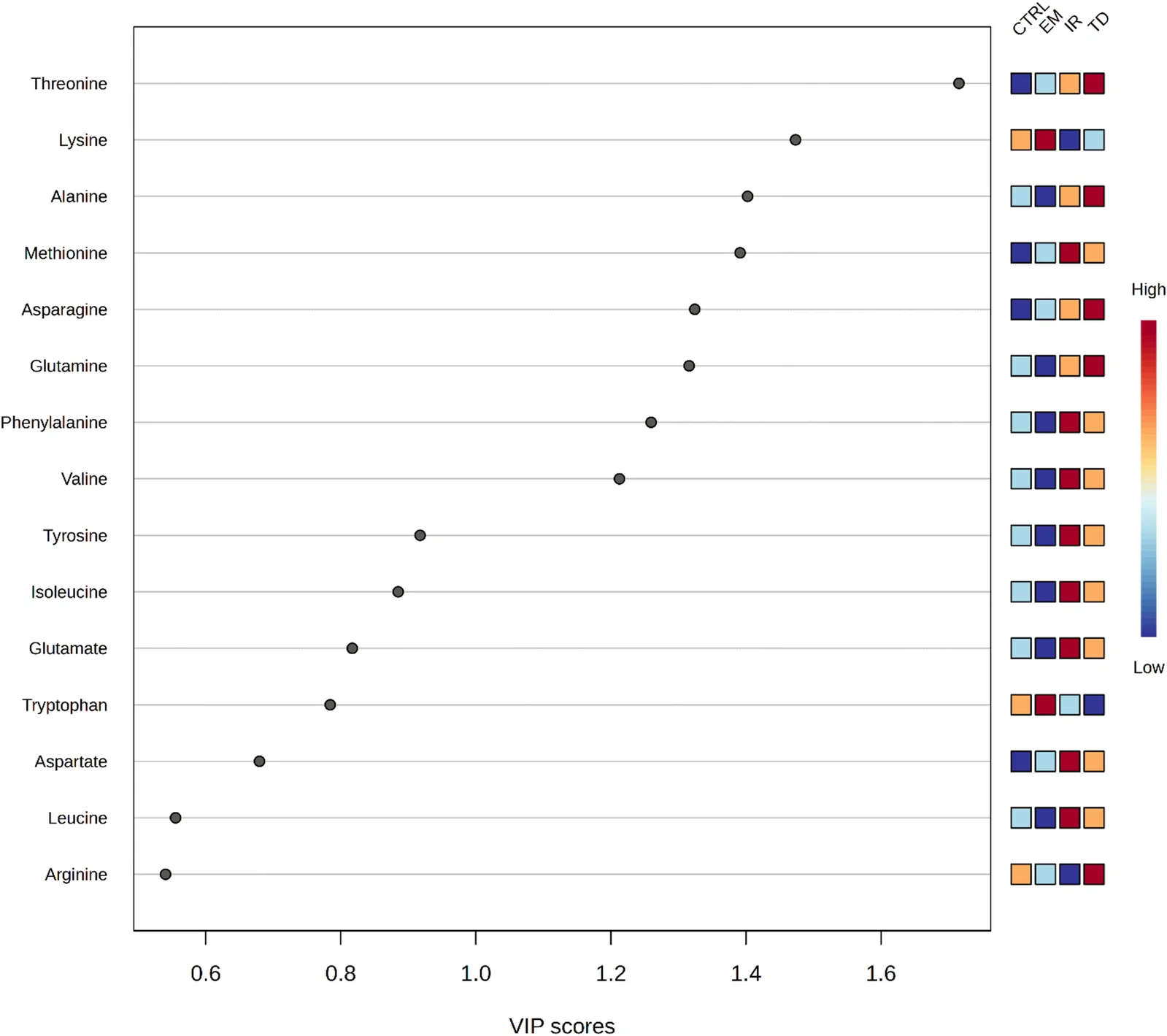
VIP scores from the PLS-DA model presenting amino acids contributing to group separation. Higher VIP scores indicate greater influence on the model. The heatmap on the right shows relative amino acid abundance across the study groups (CTRL, EM, IR, TD). Group order is consistent across all rows and relative differences can be interpreted by comparing color intensity within each row, where warmer colors indicate higher and cooler colors indicate lower relative levels

Importantly, several of the metabolites highlighted by the VIP analysis did not reach statistical significance in the univariate tests after multiple-testing correction. This finding further supports the interpretation that disease-associated metabolic differences in FF arise from coordinated shifts across multiple amino acids rather than large changes in individual metabolite concentrations (Fig. 3).

### Pathway analysis

To explore the metabolic patterns underlying the differences observed between the study groups, pathway enrichment analysis was performed using quantified amino acids.

In the comparison between the control and IR groups, pathways related to BCAA metabolism were prominently represented, including valine, leucine and isoleucine biosynthesis and degradation. It should be noted that the KEGG pathway “Valine, leucine and isoleucine biosynthesis” reflects shared enzymatic steps in the database nomenclature, the biologically relevant process in human tissues is BCAA catabolism rather than de novo biosynthesis. Additional pathways associated with amino acid metabolism, such as cysteine and methionine metabolism and the one-carbon pool by folate pathway, were also identified, suggesting alterations in amino acid utilization and related metabolic processes in the follicular environment of IR patients.

In the TD group, several pathways related to central amino acid metabolism were enriched, including alanine, aspartate and glutamate metabolism, arginine biosynthesis, and glycine, serine and threonine metabolism. Nitrogen metabolism and glyoxylate and dicarboxylate metabolism were also represented, indicating coordinated changes in pathways involved in nitrogen handling and interconnected amino acid metabolic networks.

In contrast, no pathways remained statistically significant in the EM group after multiple testing correction, which is consistent with the PCA results showing substantial overlap between EM and control samples.

Overall, these results suggest that the metabolic differences observed among the patient groups may involve coordinated alterations in amino acid metabolic pathways, particularly those related to BCAAs and central nitrogen metabolism (Table 5).

**Table 5:** Metabolic pathway enrichment analysis of amino acid alterations in FF

Valine, leucine and isoleucine biosynthesis	Total Compound	Hits	Raw *p*	−log10(*p*)	Holm adjust	FDR	Impact	Disease
	8	4	0.0013	2.8637	0.0451	0.0347	0	IR
Valine, leucine and isoleucine degradation	40	3	0.0021	2.6767	0.0674	0.0347	0	IR
Pantothenate and CoA biosynthesis	20	3	0.0116	1.9347	0.3603	0.0833	0	IR
Cysteine and methionine metabolism	33	3	0.0119	1.925	0.3603	0.0833	0.2222	IR
One carbon pool by folate	26	4	0.0127	1.8979	0.3669	0.0834	0.1071	IR
Alanine, aspartate and glutamate metabolism	28	5	0.0064	2.196	0.1847	0.0420	0.5345	TD
Arginine biosynthesis	14	4	0.0112	1.9499	0.3142	0.0498	0.2021	TD
Nitrogen metabolism	6	2	0.0115	1.9411	0.3142	0.0498	0	TD
Glycine, serine and threonine metabolism	33	4	0.0136	1.8667	0.3398	0.0498	0.4744	TD
Glyoxylate and dicarboxylate metabolism	32	4	0.01605	1.7946	0.38509	0.05295	0.11	TD

Pathways were identified using MetaboAnalyst based on detected amino acids and mapped to the KEGG database

The table shows pathway size (Total Compound), the number of matched metabolites (Hits), enrichment significance (raw *p*-value, Holm-adjusted *p*-value and FDR), and pathway impact values

### Group-specific patterns of FF amino acid profiles

**Fig. 4:**
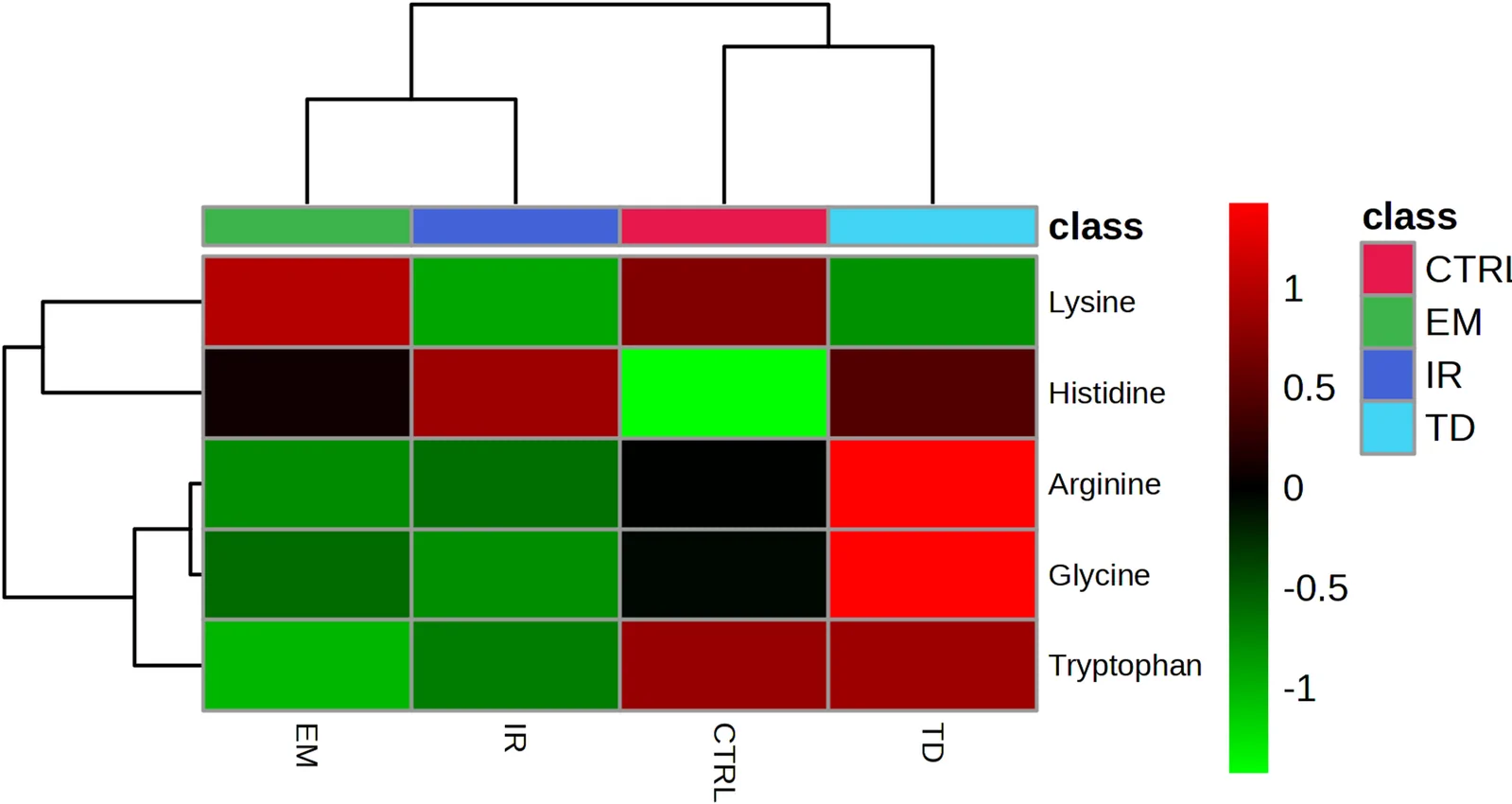
Hierarchical clustered heatmap of group-averaged concentrations of the top-ranked amino acids in FF. Amino acids were selected based on the convergence of Random Forest model and confirmed by GZLM analysis (glycine, arginine, histidine, tryptophan, lysine). Rows represent individual amino acids, columns represent study groups (CTRL, EM, IR, TD). Color scale: red indicates higher relative concentration, green indicates lower relative concentration

A hierarchical clustered heatmap was generated to visualize relative differences in FF amino acid levels across the study groups, focusing on the top-ranked amino acids (glycine, arginine, histidine, tryptophan and lysine) identified by the Random Forest model and confirmed by GZLM analysis. The results of this analysis are presented in Fig. 4.

Hierarchical clustering revealed two main breanches: one comprising the EM and IR groups and another grouping the CTRL and TD cohorts. The EM group was characterized by elevated lysine levels, while arginine, glycine and tryptophan concentrations were relatively lower compared to other groups. The IR group displayed similarly lower abundance of arginine, glycine and tryptophan, except for histidine which showed markedly higher relative values. In contrast, the TD group showed higher relative abundance of arginine and glycine alongside lower relative values of lysine. The CTRL group was characterized by higher relative relative abundance of lysine and tryptophane. 

### Diagnostic potential of amino acid profiles

**Fig. 5:**
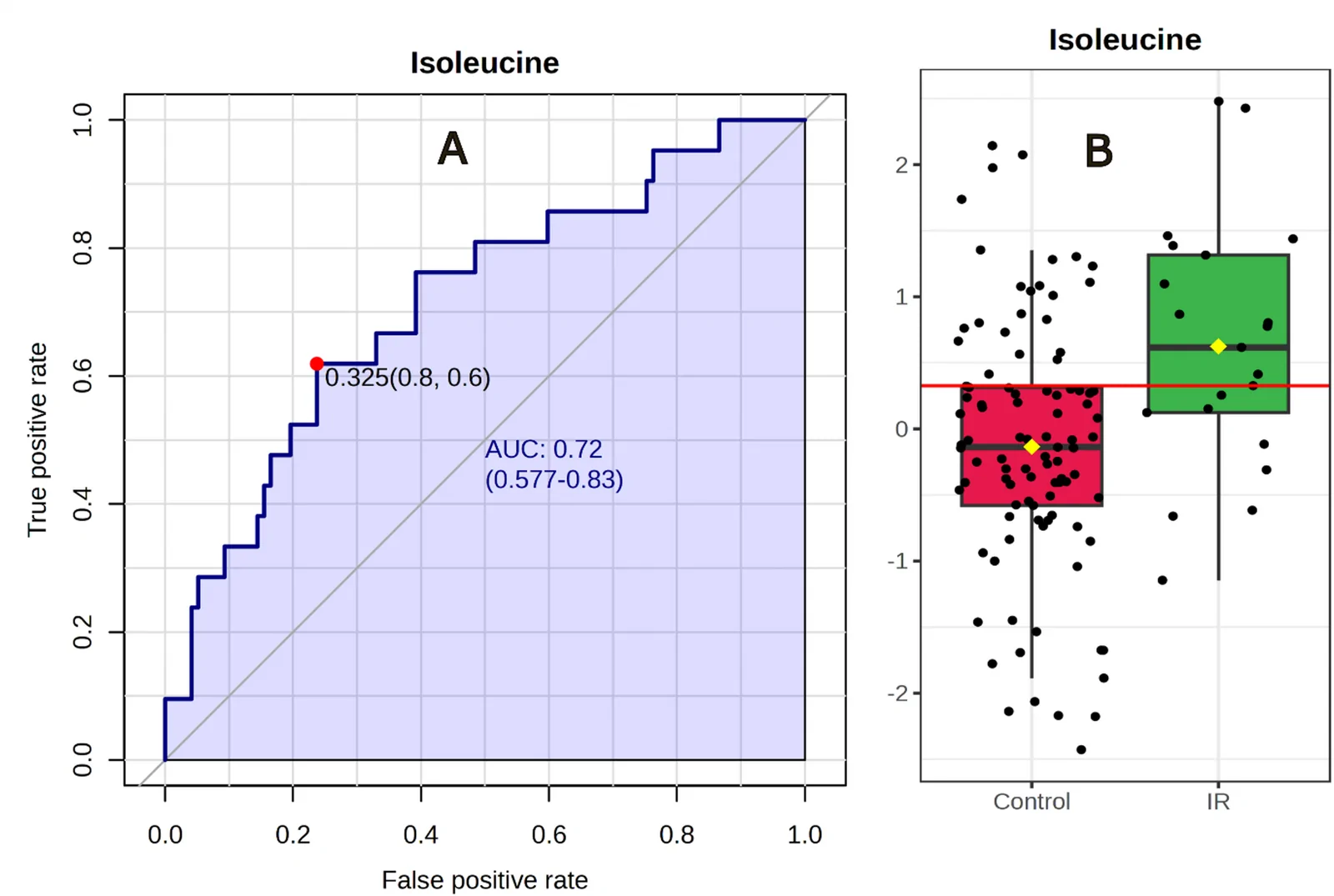
Hierarchical clustered heatmap of group-averaged amino acid concentrations in FF. Biomarker analysis of follicular fluid amino acids using ROC curve analysis. **A** The ROC curve demonstrates the discriminatory performance of isoleucine between CTRL and IR groups, which exhibited the highest diagnostic potential among the analyzed amino acids (AUC > 0.7). **B** Boxplot showing group differences in isoleucine concentrations between the CTRL and IR groups

ROC analysis was performed to evaluate the potential of individual amino acids to discriminate between disease groups and controls (Supplementary Table 2). Among the analyzed metabolites, isoleucine demonstrated the highest diagnostic performance in comparison between the IR and control groups, with an area under the curve (AUC) exceeding (AUC = 0.72), indicating moderate discriminative ability (Fig. 5). None of the other amino acids reached this threshold in the EM and TD comparisons. These findings are consistent with the pathway analysis results, which showed alterations in the BCAA metabolism in the IR group.

To further explore the discriminative performance of the amino acid profile, a Random Forest-based feature ranking was performed. Isoleucine and valine emerged as the most influential variables in discriminating IR patients from controls, followed by glycine and arginine. Although multivariate ROC analysis yielded a moderate predictive accuracy (maximal AUC = 0.602 with 20 features), the high importance scores of these specific amino acids consistently support the findings of the adjusted regression models. This cross-validation between frequentist statistics (GZLM) and machine learning (Random Forest) reinforces the significance of the BCAA and urea cycle disruptions in the follicular environment of IR patients.

In the EM group, the metabolic alterations were more subtle but highly specific. Both the GZLM and the Random Forest classification identified histidine as the primary marker of the disease, with a significant independent elevation compared to controls (*p* = 0.039). Although the multivariate ROC analysis showed limited diagnostic power (AUC range 0.52–0.54), the consistent selection of histidine as the top-ranking feature (importance score: 0.76) suggest a targeted disruption of the histidine-histamine pathway in the FF of women with endometriosis, rather than a broad amino acid dysregulation.

Finally, patients with thyroid disorders demonstrated a complex metabolic shift in the follicular environment. The multivariate ROC analysis for the TD group yielded an AUC of 0.609, further suggesting that endocrine disruptions do not rely on a single biomarker but rather induce a systemic reconfiguration of the follicular amino acid pool. Across all disease groups (EM, IR and TD), the consistency between frequentist statistics and machine learning feature ranking confirms that these amino acid signatures are reliable indicators of the altered oocyte microenvironment, independent of maternal BMI.

Overall, these analyses demonstrate that reproductive and endocrine disorders are associated with subtle but coordinated alterations in the amino acid composition of FF, reflecting disease-specific metabolic signatures within the follicular microenvironment.

### Correlation between FF amino acid concentrations and IVF parameters

To address the potential relationship between FF amino acids profiles and individual-level IVF outcomes, Spearman correlation analyses were performed between the concentrations of all 20 amino acids and key clinical parameters, including the number of retrieved oocytes, mature oocytes (MII), fertilized oocytes and number of blastocysts. False discovery rate correction was applied separately for each outcome variable using the Benjamini-Hochberg method. The results are presented in Supplementary Table 1.

After FDR correction, the majority of amino acids showed consistent and significant negative correlations with the outcome parameters. The strongest and most consistent associations were observed arginine, alanine, tyrosine, valine, methionine, leucine and lysine, which remained significant across multiple outcome variable after correction. In contrast, threonine and cysteine showed significant associations with fewer outcome parameters compared to the other amino acids.

The consistently negative direction of these associations may reflect the active metabolic uptake of amino acids by competent oocytes and surrounding granulosa cells, though this interpretation requires further investigation. These results indicate that higher FF amino acid concentrations are associated with lower IVF outcome parameters, a pattern that is consistent across the majority of the measured amino acids and robust to multiple testing correction.

## Discussion

This study provides a comparative analysis of FF amino acid profiles across multiple infertility-associated conditions, such as IR, EM and TD, within a unified analytical framework. One of the key findings of this study is that, while the global amino acid composition of FF remains broadly conserved, disease-specific alterations emerge at the level of coordinated metabolic shifts rather than larger individual changes. Importantly, these differences are relatively small and distributed across multiple metabolites, rather than reflecting strong or clearly separated group-specific metabolic phenotypes. This pattern suggests that the follicular environment is not metabolically static but instead undergoes fine-tuned reprogramming in response to systemic pathophysiological states. Such small but structured alterations are consistent with the tightly regulated nature of the oocyte microenvironment [18], where even small metabolic deviations may have functional consequences.

Although the detected group differences showed statistical significance, their overall magnitude was modest, as also indicated by the PERMANOVA effect size and the substantial overlap observed in PCA. Because of this the identified alterations should be interpreted as moderate shifts in metabolic leaning rather than pronounced metabolic reprogramming.

Despite this overall stability, disease-specific metabolic alterations were detected. Among the investigated conditions, IR exhibited a characteristic metabolic signature marked by significantly decreased glycine and arginine levels and enrichment of BCAA-related pathways. These findings align with known systemic metabolic features of IR [19] and extend them to the ovarian microenvironment [20]. The reduction in glycine, a key component of cellular redox balance [21], may indicate an altered metabolic state that could influence oocyte quality through impaired antioxidant capacity [22]. In parallel, the involvement of BCAA metabolism suggests a broader reorganization of energy-related pathways within the follicles [23]. However, it is important to emphasize that these interpretations are based on associative metabolic patterns and do not provide direct mechanistic evidence. Thus, while IR appears to be linked to a distinct metabolic tendency in FF, the functional implications remain to be clarified in future studies.

In contrast, TD was associated with a broader and more pronounced remodeling of the amino acid landscape, including decreased histidine, tryptophan and lysine concentrations. Given the central role of thyroid hormones in regulating systemic metabolism [24], these findings suggest that thyroid-related endocrine disturbances may exert widespread effects on the biochemical composition of the FF. The observed changes in FF amino acid composition may reflect altered metabolic processes within the follicular microenvironment conditions of thyroid dysfunction. Tryptophan metabolism has been implicated in reproductive physiology due to its role in immune regulation and oxidative stress through pathways such as the kynurenine pathway [25, 26]. Similarly, histidine participates in antioxidant defense [27] and can serve as a precursor for histamine synthesis [28] which is involved in inflammatory and vascular responses within reproductive tissues. Together, these findings suggest that TD may induce a systemic metabolic shift that also affects the biochemical environment of ovarian follicle.

In contrast to the broader metabolic changes observed in IR and TD patients, the metabolic alterations associated with EM appeared more selective and localized with histidine emerging as a key altered metabolite. EM is characterized by chronic inflammation and oxidative stress within the pelvic environment [29] and alterations in histidine metabolism may reflect inflammatory activity within the follicular compartment [30]. Histidine plays a role in immune regulation and vascular permeability, processes that are known to be dysregulated in EM [31]. Unlike IR and TD, which represent systemic metabolic conditions, EM may primarily influence the follicular microenvironment through localized, inflammation-driven mechanisms, resulting in a more targeted metabolic signature.

Importantly, the consistency between univariate statistics, BMI-adjusted models and multivariate feature ranking strengthens the robustness of these findings. However, the supervised models (PLS-DA and Random Forest) demonstrated limited predictive performance and should therefore be regarded primarily as exploratory tools rather than confirmatory evidence. A focused heatmap based on the top-ranked amino acids identifies by the convergence of Random Forest and GZLM analyses further supported the disease-specific patterns, with the IR and EM groups forming one cluster and the CTRL and TD groups another, reflecting diagnosis-dependent differences in the FF amino acid microenvironment. The modest discriminative performance of individual amino acids and multivariate models reflects the distributed, multi-metabolite nature of disease-associated alterations in FF and underscores that the clinical utility of FF amino acid profiling will likely depend on the integration of multiple metabolic features rather than individual biomarkers.

In conclusion, the study demonstrates that infertility-associated conditions are linked to small but coordinated alterations in the amino acid profile of the FF. These findings should be interpreted as associative and exploratory, highlighting trends in metabolic regulation rather than definitive mechanistic information. Future studies integrating multi-omics approaches and functional validation will be essential to further elucidate the role of these metabolic changes in reproductive outcomes.

## Limitations

Several limitations should be considered. The relatively small sample sizes of the disease groups, particularly in EM and IR groups, may have limited the statistical power to detect additional subtle metabolic differences. The TD group was not further subclassified by etiology (e.g., Hashimoto’s thyroiditis vs. subclinical vs. overt hypothyroidism), which may have introduced heterogeneity within this group and potentially attenuated disease-specific metabolic signals. Future studies with larger, well-characterized cohorts and more granular disease subclassification and functional validation are warranted to validate and extend present findings. Furthermore, the lack of complementary functional measurements restricts mechanistic interpretation. Nevertheless, the consistent patterns observed across multiple analytical approaches support the validity of the identified metabolic signatures. As an observational metabolomics study, this present work was designed to identify and characterize disease-associated amino acid signatures rather than to elucidate underlying mechanisms. The identified alterations should therefore be regarded as hypothesis-generating findings that provide a basis for future functional and mechanistic investigations, including in vitro models of follicular cell metabolism.

## Conclusion

Infertility-associated conditions are linked to distinct, diagnosis-specific alterations in the amino acid composition of follicular fluid, reflecting coordinated metabolic reprogramming of the ovarian microenvironment. These changes are characterized by pathway-level patterns rather than single metabolite biomarkers. Our findings highlight the sensitivity of the follicular environment to systemic metabolic and endocrine disturbances and support the relevance of multi-metabolite signatures in understanding reproductive dysfunction. Further studies integrating multi-omics approaches are warranted to clarify their biological and clinical significance. Independent validation in larger and clinically more homogeneous cohorts will be necessary before translational conclusions can be drawn.

## Supplementary Information


Supplementary Material 1.



Supplementary Material 2.



Supplementary Material 3.


## Data Availability

The datasets used and/or analysed during the current study are available from the corresponding author on reasonable request.

## Abbreviations

AUC: Area under curve
ART: Assisted reproductive technology
BMI: Body mass index
BCAA: Branched-chain amino acids
EM: Endometriosis
FDR: False discovery rate
FF: Follicular fluid
GZLM: Generalized linear models
HOMA-IR: Homeostatic model assessment of insulin resistance
IR: Insulin resistance
IVF: In vitro fertilization
KEGG: Kyoto Encyclopedia of Genes and Genomes
PLS-DA: Partial least squares-discriminant analysis
PERMANOVA: Permutational multivariate analysis of variance
PCA: Principal component analysis
RF: Random Forest
ROC: Receiver operating characteristics
TD: Thyroid dysfunction
UHPLC: Ultra-high-performance chromatography
VIP: Variable importance in projection
